# Neuroprotective properties of zinc oxide nanoparticles: therapeutic implications for Parkinson's disease

**DOI:** 10.1042/BSR20241102

**Published:** 2024-11-06

**Authors:** Kim San Tang, Wesley Zhi Chung See, Rakesh Naidu

**Affiliations:** 1 School of Pharmacy, Monash University Malaysia, Jalan Lagoon Selatan, 47500 Bandar Sunway, Selangor, Malaysia; 2 Jeffrey Cheah School of Medicine and Health Sciences, Monash University Malaysia, Jalan Lagoon Selatan, 47500 Bandar Sunway, Selangor, Malaysia

**Keywords:** amyloid, apoptosis, nanoparticles, oxidative stress, Parkinsons disease, zinc oxide

## Abstract

Parkinson's disease (PD) significantly affects millions of people worldwide due to the progressive degeneration of dopamine-producing neurons in the substantia nigra pars compacta. Despite extensive research efforts, effective treatments that can halt or reverse the progression of PD remain elusive. In recent years, nanotechnology has emerged as a promising new avenue for addressing this challenge, with zinc oxide nanoparticles (ZnO-NPs) standing out for their extensive therapeutic potential. ZnO-NPs have shown remarkable promise in neuroprotection through several key mechanisms. The multifaceted properties of ZnO-NPs suggest that they could play a crucial role in intervening across various fundamental mechanisms implicated in PD. By targeting these mechanisms, ZnO-NPs offer new insights and potential strategies for managing and treating PD. This review aims to provide a thorough examination of the molecular mechanisms through which ZnO-NPs exert their neuroprotective effects. It highlights their potential as innovative therapeutic agents for PD and outlines directions for future research to explore and harness their full capabilities.

## Introduction

There is growing recognition that neurodegenerative diseases are becoming increasingly prevalent among the ageing population [1]. Neurodegeneration is a progressive debilitating condition resulting from irreversible progressive degeneration and loss of neuronal networks [2]. Among these disorders, Parkinson's disease (PD) stands as one of the most prevalent and debilitating, affecting millions globally [3]. PD is characterized by the irreversible degeneration and loss of crucial neuronal networks within the central nervous system (CNS), mainly affecting dopamine-producing neurons in the substantia nigra pars compacta [4]. As these neurons deteriorate, patients experience a spectrum of motor and non-motor symptoms, including tremors, rigidity, bradykinesia, and cognitive impairment, profoundly affecting their quality of life [5]. Despite extensive research efforts, effective treatments capable of halting or reversing PD's relentless progression remain elusive, leaving patients and caregivers grappling with its debilitating consequences.

In recent years, nanotechnology has emerged as a promising avenue, offering innovative approaches to combat neurodegeneration [6]. Nanoparticles, which are colloidal particles ranging from 1 to 100 nm in size, have emerged as a promising approach for this purpose [7]. Among the myriad nanomaterials currently under investigation, zinc oxide nanoparticles (ZnO-NPs) have garnered significant attention due to their versatility and demonstrated efficacy across multiple domains. Research has shown that ZnO-NPs exhibit anti-cancer properties, making them potential candidates for targeted therapies [8,9]. Additionally, their anti-microbial effects can help combat infections, while their antioxidant capabilities contribute to reducing oxidative stress, a key factor in neurodegenerative diseases [8,10,11]. Furthermore, ZnO-NPs possess anti-apoptotic and anti-inflammatory properties, allowing them to mitigate cell death and inflammation, the two critical processes implicated in neurodegeneration [11–13]. This multifaceted utility positions ZnO-NPs as a particularly attractive option in the quest for effective interventions against neurodegenerative disorders.

ZnO-NPs are synthesized using various methods, including sol-gel processes and hydrothermal synthesis, allowing for precise control over their size and shape [14]. Their unique physicochemical characteristics, including a high surface area-to-volume ratio and intrinsic antibacterial properties, enhance their reactivity and potential for interaction with biological molecules [15]. These attributes make ZnO-NPs particularly promising for addressing PD pathology, as they can mitigate oxidative stress, improve stability and delivery of neuroprotective agents, and modulate inflammatory responses. Furthermore, ZnO-NPs also exhibit multifaceted neuroprotective properties, holding promise as a potential breakthrough in PD management [16]. ZnO-NPs, owing to their unique physicochemical characteristics, exert diverse effects on various pathological mechanisms underlying neurodegeneration, offering new insights into the complex aetiology of PD and the development of targeted interventions [17].

This review aims to explore the ability of ZnO-NPs to modulate key pathological pathways underlying PD, including neurotrophic factor expression, inhibit fibril formation of aggregation-prone protein, reduce oxidative stress, suppress inflammation, and mitigate neuronal apoptosis. The neuroprotective effects of ZnO-NPs are summarized in Table 1. By elucidating the mechanisms through which ZnO-NPs exert their neuroprotective effects, this review seeks to provide a comprehensive understanding of their therapeutic relevance in PD and lay the groundwork for future research directions.

**Table 1:** Neuroprotective properties of zinc oxide nanoparticles

Disease model	Induction	Experimental unit	Doses of ZnO-NPs	Administration route	Treatment duration	Effects	References
Cerebral ischemia	BCCAO (12–15 min)	Fischer rats, male, 8–10 weeks old, ∼260 g.	10 mg/kg b.w.	i.p., post BCCAO	every alternate day up to day 6	↑ neurobehavior and synaptic plasticity; ↑ Neurabin-2, NT-3 (brain tissue)	Barui *et al.*, 2020 [18]
		Neuro 2a cells	10 µg/mL	Cell culture	48 h	↑ neurogenic activity; ↑ BDNF mRNA	Barui *et al.*, 2020 [18]
		Zebrafish embryos	5 µg/mL	In vitro	24 h	↑ BDNF, GDNF, NGF, NT-3, VEGF	Barui *et al.*, 2020 [18]
Chemotherapy- induced neurotoxicity	Cisplatin, (5 mg/kg/week, i.p.) for four weeks	Wistar rats, male, 250–270 g	5 mg/kg/ day	i.p.	4 weeks	Hippocampus: ↑ SOD, CAT, GPx, GSH, GSH/GSSG ratio; ↓ MDA, GSSG; ↑ BDNF, NGF	Erfani Majd *et al.*, 2021 [20]
AD, PD, CJD, Prion-associated diseases		Insulin amyloid	80 µM	In vitro	4, 10, and 24 h	↑ Amyloid degradation	Girigoswami *et al.*, 2019 [33]
AD, PD, Prion-associated diseases		HEWL amyloid	10 µM	In vitro	120 h	↓ Amyloid fibrillation	Ban *et al.*, 2016 [34]
PD	6-OHDA (50 or 100 µM)	SH-SY5Y cells	0.081 and 0.814 µg/mL	Cell culture	24 h	↓ ROS, p53, cell death	Pan *et al.*, 2020 [47]
PD	6-OHDA (100 µM)	SH-SY5Y cells	10, 30, and 50 µg/mL	Cell culture	24 h	↓ ROS; ↑ MMP; ↓ apoptotic cell death	Ding *et al.*, 2020 [48]
PD	Rotenone, (60 mg/kg, oral) for 10 days	Wister rats, male, 200–211 g	10 mg/kg b.w.	Oral	10 days	Brain tissue: ↑ SOD, CAT, AChE; ↓ MDA, NO	Akintunde *et al.*, 2021 [49]
Type 2 diabetes-induced neurotoxicity	HFD for 8 weeks, followed by STZ (i.p.)	Wistar rats, male, 8–10 months, 120–150 g	10 and 50 mg/kg/day	Oral	6 weeks	↑ learning and memory; Hippocampus: ↓ histological lesions; ↓ inflammation; ↓ MDA, NO; ↑ CAT, SOD, GPx, GST, GSH; ↓ Bax; ↑ Bcl-2; ↓ p-p38 MAPK, p-MEK, pERK1/2, p-Tau; ↓ Aβ-42, APP, BACE-1; ↑ BDNF, ADAM-10	Abdulmalek *et al.*, 2021 [31]
MSG-induced neurotoxicity	MSG, 17.5 mg/kg b.w.	Rats, male, 200–250 g	10 mg/kg b.w.	Oral	Samples were collected after 30 days of treatment.	Brain tissue: ↑ dopamine, adrenaline, noradrenaline, ↑ AChE, thiol; ↑ SOD, CAT, GSH, GPx, MPO, XO, BDNF; ↓ COX-2, PGE2; ↓ histological lesions	Hamza *et al.*, 2019 [51]
Lead-induced neurotoxicity	Lead oxide, 10%	Wistar rat, male, 150–190 g	6%, cream, daily	Topical	2 weeks	Brain tissue: ↓ histological lesions; ↓ MDA, BAX, COX-2, caspase-3; ↑ CAT, GSH	Hassanen *et al.*, 2021 [53]
Spinal cord injury	Axon axotomy	Primary cortical neurons from wild-type Sprague–Dawley E18 embryonic rats	5 µg/mL	In vitro	24 h	↑ SOD1, SOD2; ↑ MMP, BAX, BCL-2; ↓ Bax/Bcl-2; ↑ IL-4, IL-10; ↑ PI3K-Akt	Liu *et al.*, 2022 [60]

Abbreviations: 6-OHDA, 6-hydroxydopamine; AChE, acetylcholinesterase; AD, Alzheimer's disease; ADAM-10, A Disintegrin and metalloproteinase domain-containing protein 10; APP, amyloid precursor protein; Aβ-42, amyloid-beta 42; b.w., body weight; BACE-1, beta-site APP cleaving enzyme 1; BAX, BCL-2-associated X protein; BCCAO, bilateral common carotid artery occlusion; BDNF, brain-derived neurotrophic factor, CAT, catalase; CJD, Creutzfeldt-Jakob disease; COX-2, cyclooxygenase 2; GDNF, glial cell line-derived neurotrophic factor; GPx, glutathione peroxidase; GSH, glutathione, GSSG, glutathione disulphide; GST, glutathione S-transferase; HEWL, hen egg white lysozyme; HFD, high-fat diet; i.p., intraperitoneal; IL, interleukin; MDA, malondialdehyde; MMP, mitochondria membrane potential; MPO, myeloperoxidase; MSG, monosodium glutamate; NF-κB, nuclear factor-kappa B; NGF, nerve growth factor; NO, nitric oxide; NT-3, neurotrophin-3; PD, Parkinson's disease; PGE2, prostaglandin E2; PI3K, phosphoinositide 3-kinase; ROS, reactive oxygen species; SOD, superoxide dismutase; TNF-α, tumour necrosis factor alpha; VEGF, vascular endothelial growth factor; XO, xanthine oxidase.

## ZnO-NPs increase neurotrophic factor expression

ZnO-NPs have been observed to exert neuroprotective effects through various mechanisms. For instance, a study by Barui *et al*. [18] demonstrated that exposure of Neuro 2a cells to ZnO-NPs (10 μg/mL, 24 h) led to a 2-fold increase in the mRNA expression of brain-derived neurotrophic factor (BDNF). Lowered BDNF levels were observed in patients with PD [19]. Another study using chemically and green ZnO-NPs from *Aloe vera* leaf extract showed up-regulation of BDNF and nerve growth factor (NGF) mRNA expression in the hippocampus of mice treated with cisplatin, a neurotoxic chemotherapeutic agent [20]. It has been shown that activation of the NGF signalling pathway protects PC12 cells from toxicity induced by the dopaminergic neurotoxin MPP^+^ [21]. Neurotrophic factors play a vital role in synaptic plasticity, neuronal growth, survival, differentiation, and maturation in the CNS [22].

Vascular endothelial growth factor (VEGF) plays a crucial role in promoting the survival of DA neurons treated with 6-hydroxydopamine (6-OHDA), a widely used synthetic neurotoxin in PD research [23]. This neuroprotective effect underscores the importance of growth factors in mitigating neuronal damage, a role that ZnO-NPs could potentially enhance by facilitating the VEGF signalling pathway. Additionally, a recent study using an adult zebrafish PD model indicated that cellular localization of neurotrophin-3 (NT-3) in the brains protects against MPTP-induced down-regulation of *bdnf* gene and loss of locomotor function [24]. ZnO-NPs may aid in the localization and stability of NT-3, thereby helping to preserve BDNF levels and promote neuronal health in PD contexts. In a rat model of PD, levels of glial cell-derived neurotrophic factor (GDNF) were found to be reduced, highlighting the need for neuroprotective strategies [25].

Interestingly, Barui *et al*. [18] reported that zebrafish embryos exposed to ZnO-NPs (5 μg/mL, 24 h) exhibited up-regulation of mRNA expression of various neurotrophic factors, including VEGF, BDNF, NT-3, NGF, and GDNF [18]. Moreover, Neuro 2a cells treated with ZnO-NPs (10 μg/mL, 48 h) demonstrated a 1.5-fold increase in the neurite length, indicating the neuritogenic activity of ZnO-NPs [18]. Consistent with this, administration of ZnO-NPs (10 mg/kg, i.p., every other day up to day 6) in rats with global cerebral ischemia resulted in enhanced sensorimotor function and locomotion, as well as improved dendritic spine density and synaptic connection in cortical neurons [18]. These findings suggest that ZnO-NPs can potentially promote neuritogenic activity and exert neuroprotective effects at both cellular and *in vivo* levels. Nonetheless, the regulation of neurotrophic factors by ZnO-NPs has yet to be further explored specifically in PD models.

## ZnO-NPs inhibit amyloid fibrillar formation and tau phosphorylation

Cognitive decline is a common non-motor symptom of PD. It has been reported that the majority of PD patients (∼80%) who live for more than 10 years will develop dementia [26]. Deposition of amyloid beta (Aβ) in the brain has been associated with cognitive impairment in Alzheimer's disease (AD) and PD. Amyloid accumulation in PD is an area of growing research, particularly in the context of cognitive impairment and dementia associated with PD. While Aβ plaques are primarily linked to AD, some studies have detected these plaques in the brains of individuals with PD, especially those experiencing cognitive decline [27–29]. The soluble monomer proteins and peptides undergo a transformation into highly organized fibrillar amyloid protein aggregates that accumulate in the brain and other tissues [30]. The deposition of these fibrillar protein aggregates or amyloid in neurons disrupts protein degradation pathways, intracellular transport, and vital cell functions, contributing to neuronal loss [30]. A study by Abdulmalek *et al*. [31] highlighted that ZnO-NPs ameliorated the increase of Aβ-42 levels in the brains of rats with type 2 diabetes-induced neurodegeneration.

Numerous studies have implicated these amyloid fibrils or plaques containing highly structured cross-β sheet formation [30]. One primary strategy for neuroprotection involves inhibiting the self-assembly of the proteins responsible for fibril formation. The physicochemical differences between soluble monomer proteins and larger protein aggregates such as oligomers and fibrils can be exploited to achieve this inhibition. Additionally, it has been shown in several experimental studies that oligomers exhibit the highest level of neurotoxicity, making their blocking by ZnO-NPs a significant focus from a biological standpoint [32]. The ability of nanoparticles to form and inhibit amyloid fibrils has been investigated using various techniques, including thioflavin T (ThT)-binding assay and electron microscopy.

Girigoswami *et al*. [33] used a ThT-binding assay to study the interaction between ZnO-nanoflowers and insulin amyloid fibrils and observed a 60% decrease in fibril growth after exposure to ZnO-nanoflowers (80 μM, 24 h). They also noted an 85% reduction in fibril size following incubation with ZnO-nanoflowers (80 μM, 24 h). Another study by Ban *et al*. [34] examined the effect of three different forms of ZnO-NPs (uncapped, starch-capped, and self-assembled) on the amyloid growth of hen egg white lysozyme (HEWL). Uncapped ZnO-NPs, which lack surface modifications, exhibited higher reactivity but potentially greater toxicity. In contrast, starch-capped ZnO-NPs, coated with starch for enhanced stability and biocompatibility, showed the most significant inhibitory effect on amyloid growth. Self-assembled ZnO-NPs, organized into specific structures, offered unique properties that also contributed to the inhibition of fibrillar amyloid growth. The authors observed a dose-dependent inhibition of amyloid with all three forms of ZnO-NPs (1–20 μM, 120 h), with reductions in cross-β sheet content of amyloid fibrils of 16%, 24%, and 43% for uncapped, self-assembled, and starch-capped ZnO-NPs, respectively. Additionally, HEWL amyloid exhibited high toxicity to Neuro 2a and HaCaT cells, but this toxicity was reduced after incubation with all three types of ZnO-NPs, particularly with the starch-capped form. These findings suggest that ZnO-NPs can effectively inhibit amyloid fibrillation and mitigate amyloid-mediated cellular toxicity.

Beta-secretase 1, also known as beta-site amyloid precursor protein cleaving enzyme 1 (BACE1), is the enzyme responsible for sequential cleaving of amyloid precursor protein (APP), resulting in Aβ generation. It has been reported that phosphorylation of APP by mutant LRRK2 promotes neurodegeneration in PD [35]. BACE1 and A Disintegrin and metalloproteinase domain-containing protein 10 (ADAM-10) gene polymorphisms have been associated with an increased risk of PD in different human populations [36,37]. Moreover, decreased plasma levels of ADAM10 were observed in PD patients compared with the healthy controls [37]. Abdulmalek *et al*. [31] showed that ZnO-NPs decreased gene expression of BACE1 and APP, but increased ADAM-10 in the hippocampus of diabetic rats.

Hyperphosphorylation of tau, another biomarker of AD, is also known to be involved in the pathophysiology of PD. Several post-mortem findings have shown the presence of tau in the brain of PD patients [38]. Abdulmalek *et al*. [31] also reported that daily oral exposure of diabetic rats to ZnO-NPs for six weeks, at concentrations of 10 and 50 mg/kg body weight (b.w.), reduced the level of phosphorylated tau in the hippocampus region by 50% and 80%, respectively. The pathological hallmark of PD is the presence of intracellular inclusions in the brain, named Lewy bodies [39]. Interestingly, colocalization of tau and α-synuclein has been demonstrated in Lewy bodies of PD patients [40,41]. α-Synuclein can form fibrillar aggregates that contribute to the development of PD [42]. Indeed, fibrillation of α-synuclein promotes mitochondrial dysfunction and cytotoxicity [43]. Research has indicated that ZnO-NPs might influence the aggregation of α-synuclein, potentially reducing its accumulation or promoting its clearance [44,45]. This could help mitigate the formation of Lewy bodies, which are aggregates of alpha-synuclein found in PD.

## ZnO-NPs reduce oxidative stress

A growing body of evidence suggests a strong connection between oxidative stress and the development of neurodegenerative diseases. With its high lipid content and energy demands, the brain is particularly susceptible to oxidative stress [46]. Specific populations of neurons in different brain regions are especially vulnerable to oxidative stress, leading to functional decline and cell death [46]. Research has increasingly supported the neuroprotective potential of ZnO-NPs in reducing oxidative stress. Pan *et al*. [47] investigated the protective effects of ZnO-NPs against oxidative stress induced by 6-OHDA in SH-SY5Y cells. Treatment with 6-OHDA increased reactive oxygen species (ROS) levels by 1.4-fold, but pre-treatment with ZnO-NPs (0.081 mg/mL for 24 h) reduced the ROS increase by 30%. Similarly, Ding *et al*. [48] demonstrated a 45% reduction in ROS levels induced by 6-OHDA in SH-SY5Y cells after treatment with ZnO-NPs (30 μg/mL, 24 h). In another animal model of PD, Akintunde *et al*. [49] showed a 1.2-fold increase in malondialdehyde (MDA) levels in the brain of rotenone-treated rats. However, a 10-day treatment with green ZnO-NPs (10 mg/kg b.w.) synthesized from *Moringa oleifera* leaves decreased the brain MDA levels by 30%.

Glutathione (GSH) is crucial in protecting the brain against oxidative stress, as it is highly susceptible to free radicals. Imbalances in GSH redox have been implicated in the development of various brain disorders, including autism, bipolar disorder, schizophrenia, AD, PD, amyotrophic lateral sclerosis, Huntington's disease, and multiple sclerosis [50]. Studies conducted on *in vivo* models and clinical samples of these neuropsychiatric and neurodegenerative diseases have consistently shown decreased GSH levels, increased GSH/ glutathione disulfide (GSSG) ratios, and impaired expressions and activities of enzymes involved in GSH metabolism, such as glutathione peroxidase (GPx), glutathione S-transferase and glutamate-cysteine ligase, in the blood or brain [50].

In several *in vivo* models, ZnO-NPs have demonstrated the ability to restore anti-oxidant levels to a healthy state in the brain when anti-oxidant homeostasis is disrupted. Using a streptozotocin-induced model, Abdulmalek *et al*. [31] found that ZnO-NPs (10 mg/kg/day, six weeks) attenuated oxidative stress levels by increasing GSH levels and glutathione S-transferase activity by 8.1- and 6.8-fold, respectively, in the hippocampus of Wistar rats. Hamza *et al*. [51] administered monosodium glutamate to rats, which induced oxidative stress in brain tissues, resulting in neurodegenerative injuries such as haemorrhage, necrosis, and glial cell death. They observed diminished GSH levels and GPx activity. However, treatment with ZnO-NPs (10 mg/kg for 30 days) and green tea extract increased GSH levels and GPx activity, facilitating the recovery of congested areas with normal neural fibre appearance [51]. In another study by Erfani Majd *et al*. [20], cisplatin reduced GSH levels and the GSH/GSSG ratio while increasing GSSG levels in the hippocampus of rats. Cisplatin is an anti-cancer drug that has been associated with several adverse effects, particularly neurotoxicity [52]. Treatment with green ZnO-NPs (5 mg/kg/daily, 28 days) synthesized from *Aloe vera* leaf improved GSH level by 2-fold and GSH/GSSG ratio by 8-fold compared with the control group [20].

ZnO-NPs have also been shown to up-regulate other anti-oxidant enzymes, including superoxide dismutase (SOD) and catalase (CAT), in the brain of treated animals, highlighting their potential neuroprotective effects [31,51,53]. In the context of PD, the study by Akintunde *et al*. [49] further revealed that the rats treated with neurotoxin rotenone exhibited a significant 2-fold decrease in the activities of SOD and CAT in their brains. However, the administration of ZnO-NPs at a dosage of 10 mg/kg b.w. for 10 days effectively restored SOD and CAT activity to control levels, demonstrating the ability of these nanoparticles to mitigate oxidative damage. This restoration suggests that ZnO-NPs not only counteract the detrimental effects of rotenone but also promote a healthier antioxidant environment within the brain, reinforcing their therapeutic potential in managing oxidative stress-related neurodegenerative conditions like PD.

## ZnO-NPs inhibit cell apoptosis

Extensive evidence supports the notion that neuronal cell death through apoptosis is a highly conserved process that occurs during the development and maturation of the nervous system, as well as in pathological states [54]. Studies analyzing post-mortem tissues from patients, as well as investigations using experimental animals and cell cultures, have implicated neuronal apoptosis in the pathogenesis of various neurological and neurodegenerative diseases, such as PD. Post-mortem brain tissues display characteristics of apoptotic cells, such as nuclear condensation and chromatin fragmentation [55]. Moreover, the overexpression of pro-apoptotic proteins, including BCL-2-associated X protein (BAX) and increased levels of caspase-3, -8, and -9, along with the down-regulation of the anti-apoptotic BCL-2 protein in neurons of post-mortem samples, indicate the involvement of apoptosis in the development of neurodegenerative diseases [56–59].

Numerous studies have explored the potential of ZnO-NPs as a neuroprotective agent to enhance cellular resistance to apoptotic triggers. Pan *et al*. [47] observed a 65% decrease in BAX/BCL-2 mRNA expression ratio in SH-SY5Y cells exposed to ZnO-NPs (0.814 μg/mL, 24 h ). Furthermore, ZnO-NPs (0.814 μg/mL, 24 h ) protected against neuronal cell death when challenged with 6-OHDA and H_2_O_2_ by reducing the protein expression of p53. Abdulmalek *et al*. [31] demonstrated that treatment with ZnO-NPs significantly decreased BAX mRNA expression and increased BCL-2 mRNA expression in the hippocampus and cortex of rats with type 2 diabetes-induced neurodegeneration. Specifically, BCL-2 mRNA expression increased by 14 to 16-fold, while BAX mRNA expression decreased by 70 to 80% in both regions. In another study conducted by Liu *et al*. [60], an *in vitro* spinal cord injury model was used, where primary cortical neuronal cell bodies and dendrites from embryonic rats were confined to the soma chamber of a microfluidic culture device, while axons were allowed to grow into the axon terminal chamber. Axotomy of axons was performed after their full extension into the terminal chamber, inducing oxidative stress and impaired mitochondria, ultimately leading to apoptosis. Following axotomy, a significant increase in the BAX/BCL-2 ratio was observed, but treatment with ZnO-NPs (5 μg/mL for 24 h ) successfully reduced the ratio by 2-fold. Furthermore, ZnO-NPs increased the mRNA expression of SOD1 and SOD2 and improved mitochondrial function, resulting in enhanced axon regeneration after axotomy.

## ZnO-NPs suppress neuroinflammation

Neuroinflammation has been associated with PD and many other neurodegenerative diseases [61]. The involvement of neuroinflammation in the pathogenesis of PD is thought to be linked to oxidative stress [62]. Oxidative stress induces neuroinflammation by regulating the translocation of the nuclear factor-kappa B (NF-κB) between cytosol and nucleus [63]. NF-κB is a master regulator of inflammation and is generally sequestered in an inactive state in the cytosol by inhibitor IκB proteins [63]. Following cell stimulation by oxidative stress, IκB proteins in the cytosol are rapidly phosphorylated and ubiquitinated, ultimately targeted for degradation by the 26S proteasome [63]. This results in the migration of the NF-κB into the nucleus, where NF-κB regulates the expression of pro-inflammatory mediators such as tumour necrosis factor alpha (TNF-α), interleukin (IL)-1β, IL-6, and cyclooxygenase-2 (COX-2) [64]. On the other hand, anti-inflammatory cytokines such as IL-1 receptor antagonist, IL-4, IL-10, IL-11, and IL-13 are produced to dampen inflammation and tone down an unnecessary immune response that can result in tissue damage [65].

ZnO-NPs have been observed to elicit anti-inflammatory properties by up-regulating the protein expression of cytoplasmic IκBα and down-regulating the expression of nuclear NF-κB, respectively, in stimulated human mast cell line-1 (HMC-1) [66]. Additionally, mRNA and protein expression of pro-inflammatory mediators such as TNF-α, IL-1β, and IL-6 were observed to be down-regulated in stimulated HMC-1 mast cells upon exposure to ZnO-NPs (0.1-10 μg/mL, 1 h ) [66]. Abdulmalek *et al*. [31] also showed that ZnO-NPs (50 mg/kg/day, 6 weeks) caused a reduction of ∼70% in the pro-inflammatory TNF-α and IL-6 protein levels in the hippocampus of rats with type 2 diabetes-induced neurodegeneration. In an *in vitro* spinal cord injury model, ZnO-NPs (5 μg/mL, 24 h ) induced a 3-fold enhancement in the mRNA expression of anti-inflammatory cytokines IL-4 and IL-10, while demonstrating minimal changes in the mRNA expression of TNF-α and IL-1β [60].

In the study by Akintunde *et al*. [49], it was shown that green ZnO-NPs (10 mg/kg b.w., 10 days) from *Moringa oleifera* leaves reduced nitric oxide (NO) levels by 25% in rats treated with rotenone. Additionally, in rats with type 2 diabetes-induced neurodegeneration, ZnO-NPs (50 mg/kg/day, 6 weeks) led to a significant 90% reduction in hippocampal NO levels [31]. Another study by Hassanen *et al*. [53] demonstrated that treatment of lead-intoxicated rats with ZnO-NPs (6% zinc cream, 14 days) resulted in a significant 40% decrease in the mRNA expression of COX-2 in brain tissues compared with lead-intoxicated rats without ZnO-NPs treatment. Finally, in the study by Hamza *et al*. [51], it was observed that monosodium glutamate-induced oxidative stress elevated COX-2 activity and prostaglandin E2 protein levels in the cerebral cortex of rats. However, when ZnO-NPs (10 mg/kg, 30 days) were administered in combination with green tea extract, COX-2 activity decreased by 50%, and prostaglandin E2 levels decreased by 20%.

## Conclusion

Despite the promising neuroprotective effects of ZnO-NPs, it is crucial to consider their potential neurotoxicity. Recent studies have raised concerns regarding the cytotoxic effects of ZnO-NPs, particularly at higher concentrations, which can lead to oxidative stress and neuroinflammation [67–69]. Mechanistically, the release of zinc ions from ZnO-NPs may disrupt metal ion homeostasis in neurons, potentially contributing to neurodegenerative processes [70]. Additionally, some research indicates that the size, shape, and surface modifications of ZnO-NPs can significantly influence their biocompatibility and toxicity profiles [71,72]. Understanding the balance between the therapeutic benefits and potential risks of ZnO-NPs is essential for their safe application in treating neurodegenerative diseases like PD.

**Figure 1:**
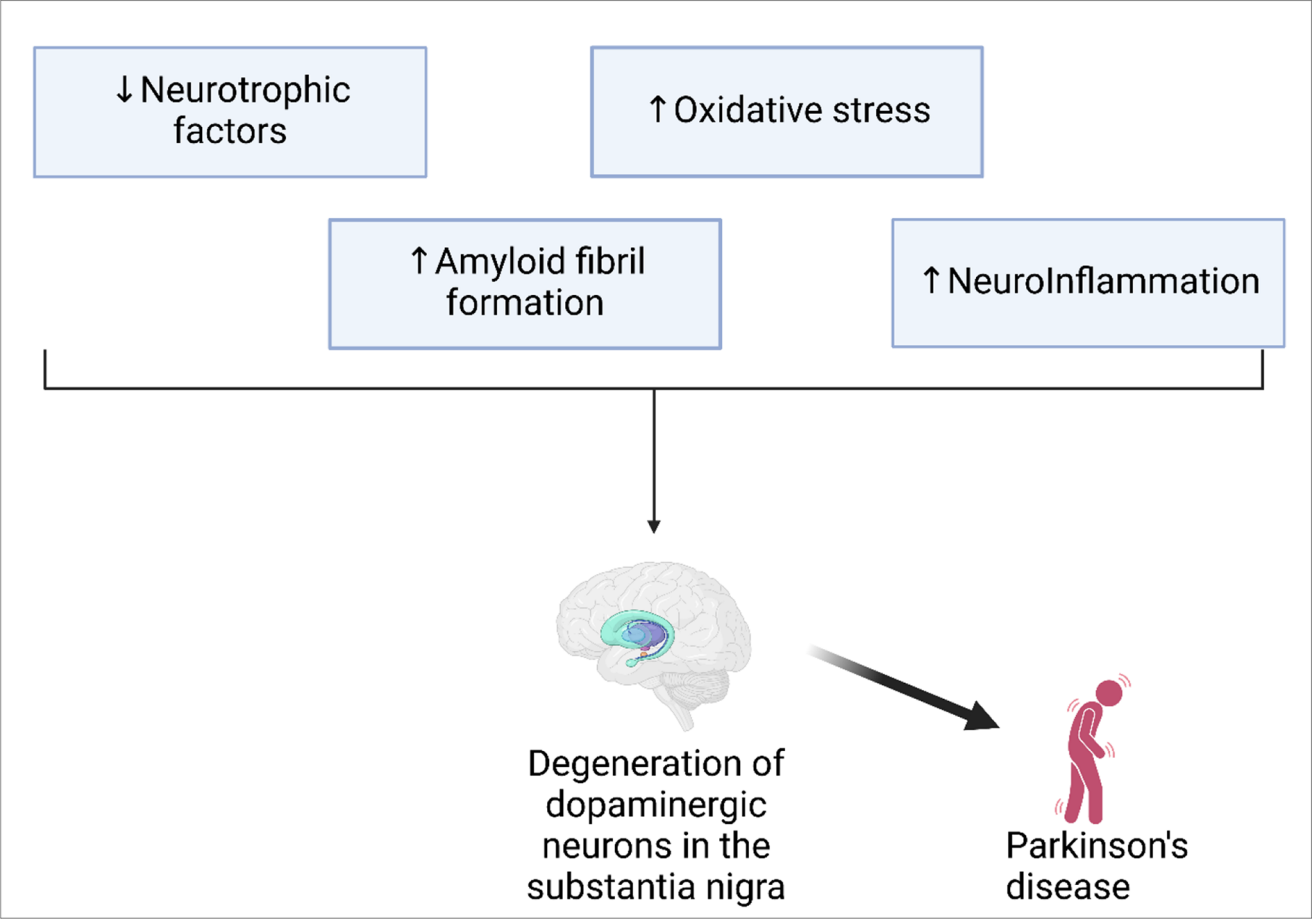
An overview of neuroprotection targets of zinc oxide nanoparticles The pathological mechanisms underlying neuronal degeneration in Parkinson's disease that could be intervened by zinc oxide nanoparticles.

In conclusion, ZnO-NPs have shown promising effects on several pathological mechanisms implicated in PD (Figure 1). Elucidating the specific molecular targets and mechanisms involved in neuroprotection by ZnO-NPs could lead to the development of more precise and effective treatments. More research work employing *in vitro* and animal models of PD should be carried out to study the neuroprotective potential of ZnO-NPs. In addition, investigating the synergistic effects of ZnO-NPs with other therapeutic compounds, such as conventional drugs or natural products, may give rise to complementary neuroprotective strategies and potentially lead to superior outcomes in PD management. Finally, future research to optimize their properties and evaluate their safety and efficacy is warranted to realize their full potential in neuroprotection and disease modification.

## Open Access

Open access for this article was enabled by the participation of Monash University in an all-inclusive *Read & Publish* agreement with Portland Press and the Biochemical Society under a transformative agreement with CAUL.

## Abbreviations

6-OHDA: 6-hydroxydopamine
AD: Alzheimer's disease
ADAM-10: A Disintegrin and metalloproteinase domain-containing protein 10
APP: amyloid precursor protein
Aβ-42: amyloid-beta 42
b.w.: body weight
BACE-1: beta-site APP cleaving enzyme 1
BAX: BCL-2-associated X protein
BDNF: brain-derived neurotrophic factor
CAT: catalase
COX-2: cyclooxygenase 2
GDNF: glial cell line-derived neurotrophic factor
GPx: glutathione peroxidase
GSH: glutathione
GSSG: glutathione disulphide
HEWL: hen egg white lysozyme
HMC-1: human mast cell line-1
i.p.: intraperitoneal
IL: interleukin
MDA: malondialdehyde
NF-κB: nuclear factor-kappa B
NGF: nerve growth factor
NO: nitric oxide
NT-3: neurotrophin-3
PD: Parkinson's disease
ROS: reactive oxygen species
SOD: superoxide dismutase
ThT: thioflavin T
TNF-α: tumour necrosis factor alpha
VEGF: vascular endothelial growth factor
ZnO-NPs: zinc oxide nanoparticles
